# Crustacean Proteases and Their Application in Debridement

**DOI:** 10.21315/tlsr2020.31.2.10

**Published:** 2020-08-06

**Authors:** Erick Perera, Leandro Rodriguez-Viera, Vivian Montero-Alejo, Rolando Perdomo-Morales

**Affiliations:** 1 Nutrigenomics and Fish Growth Endocrinology, Institute of Aquaculture Torre de la Sal, IATS-CSIC, Castellón, Valencia, Spain; 2 Center for Marine Research, University of Havana, Havana, Cuba; 3 Department of Biochemistry, Center for Pharmaceuticals Research and Development, Havana, Cuba

**Keywords:** Crustaceans, Proteases, Thermal Stability, Tropical Species, Trypsin, Debridement

## Abstract

Digestive proteases from marine organisms have been poorly applied to biomedicine. Exceptions are trypsin and other digestive proteases from a few cold-adapted or temperate fish and crustacean species. These enzymes are more efficient than enzymes from microorganism and higher vertebrates that have been used traditionally. However, the biomedical potential of digestive proteases from warm environment species has received less research attention. This review aims to provide an overview of this unrealised biomedical potential, using the debridement application as a paradigm. Debridement is intended to remove nonviable, necrotic and contaminated tissue, as well as fibrin clots, and is a key step in wound treatment. We discuss the physiological role of enzymes in wound healing, the use of exogenous enzymes in debridement, and the limitations of cold-adapted enzymes such as their poor thermal stability. We show that digestive proteases from tropical crustaceans may have advantages over their cold-adapted counterparts for this and similar uses. Differences in thermal stability, auto-proteolytic stability, and susceptibility to proteinase inhibitors are discussed. Furthermore, it is proposed that the feeding behaviour of the source organism may direct the evaluation of enzymes for particular applications, as digestive proteases have evolved to fill a wide variety of feeding habitats, natural substrates, and environmental conditions. We encourage more research on the biomedical application of digestive enzymes from tropical marine crustaceans.

**Highlights**

- The potential of digestive proteases from tropical crustaceans in debridement is revisited.
- Digestive proteases from tropical crustaceans may have advantages over their cold-adapted counterparts for this and similar uses.
- More research on the biomedical application of digestive enzymes from tropical marine crustaceans is encouraged.

## INTRODUCTION

Given their high enzyme diversity and the feasibility of large-scale fermentation, marine microorganisms are widely used in the exploration of enzyme resources for biotechnological applications (Wang *et al.* 2016; Lam *et al.* 2018) as well as in the food industry (Fernandes 2014; Maruthiah *et al.* 2016). In particular, microorganisms from extreme environments have been receiving increased attention because of the molecular features of their enzymes, which result in high catalytic activity around the boiling and freezing points of water and extreme pH values (Antranikian *et al.* 2005). Proteases are the largest group of industrial enzymes and have a variety of applications ranging from use in detergents, leather preparation and food processing. During the last decade, marine proteases of non-microbial origin have been assessed for different applications, such as the use of fish (Klomklao 2008; Shahidi & Kamil 2001; Jesus de la Cruz *et al.* 2018) and crustacean (Rossano *et al.* 2011) digestive proteases in the food industry. Conversely, digestive proteases from marine organisms have been less applied to the biomedical field.

Proteases in general are not ideal therapeutic agents; they must remain active under optimal conditions in the site of action for a period long enough to ensure adequate pharmacokinetics. Furthermore, proteases can denature, are prone to proteolytic processing, and are susceptible to inhibitors. Another drawback of proteases is their poor cell permeability, thus they cannot reach intracellular targets and are not suited for oral administration, unless intended to act on the gastro-intestinal tract (Aehle 2004). However, there are areas where the use of proteases can be employed successfully such as in topical treatments.

Proteases from a wide variety of marine organisms have been extensively studied in the context of digestion physiology, ecology and aquaculture. In contrast, only a few of these proteases have shown biomedical potential. Trypsin from Atlantic cod (*Gadus morhua*) has antipathogenic properties due to its ability to cleave proteins from viruses and bacteria and has proven to be effective in wound healing (Gudmundsdóttir *et al.* 2013; Jesus de la Cruz *et al.* 2018). Enzymes from some crustaceans have also been evaluated for wound healing (Glyantsev *et al.* 1997; Kristjansdottir & Gudmundsdottir 2000). Typically, these enzymes come from cold-adapted or temperate species and the biomedical potential of their counterparts from warm environments have received less research attention. Digestive proteases are extremely diverse in both herbivorous and carnivorous marine organisms, and have evolved to cope with a wide variety of dietary habits including detritus feeders and different kinds of scavengers. Therefore, there is still much to learn regarding the richness of digestive enzyme functionalities that occurs in marine organisms with wide-ranging feeding habits and temperature preferences.

This review aims to provide an overview of the unrealised biomedical potential of digestive proteases from marine tropical crustaceans using the debridement application as a paradigm. We discussed different approaches for debridement, the physiological role of enzymes in wound healing, the use of exogenous enzymes in debridement, and current limitations in the use of cold-adapted enzymes. We show that digestive proteases from tropical crustaceans may have advantages over their cold-adapted and temperate counterparts for these processes and for other biomedical applications as well.

## DEBRIDEMENT AS A CASE IN POINT

### Debridement

Cutaneous wound healing has been a heated topic in recent decades and has prompted the development of various therapeutic approaches (Zeng *et al.* 2018). Clinical experience strongly supports that debridement is a necessary component of wound bed preparation in chronic or hard-to-heal wounds (McCallon *et al.* 2014). Debridement is a technique aimed at removing nonviable, necrotic and contaminated tissue, until surrounding healthy tissue is exposed. Debridement enables the true extent of the wound to be understood, allows drainage of exudates and removal of dead tissue, enables a deep swab to be taken for culture and in general, encourages healing (Edmonds & Foster 2000; McCallon *et al.* 2014). Others have suggested that the main reason for debriding a wound is to avoid substratum for bacterial growth (Nano *et al.* 1996). Different approaches are currently available for debridement such as autolysis, surgical intervention, mechanical methods, biosurgery and enzymatic approaches (Gottrup 2010; Ramundo & Gray 2008; Zeng *et al.* 2018).

The common practice in wound healing includes initial and ongoing surgical or sharp debridements to remove nonviable tissue, daily saline dressing changes, off loading of pressure and systematic control of infection (Eneroth & van Houtum 2008). Low-frequency ultrasound has been tested as an alternative to surgical wound debridement (Herberger *et al.* 2011). Another mechanical method is called “wet-to-dry” in which the wound is soaked in saline to moisten hard material before the application of a moist gauze pad over the affected area. When the dressing is changed, the attached devitalised tissue is pulled free. Unlike a cloth, some materials such as monofilament polyester fibres are able to integrate devitalised tissue and debris within their structure (Bahr *et al.* 2010). Both sharp and wet-to-dry debridements are inexpensive but may remove granulating tissue and are painful for the patient.

Nonsurgical methods of debridement have received attention during the recent, as they promote the body’s own immune system to break down and digest necrotic debris (i.e. autolytic debridement) (McCallon *et al.* 2014). These methods involve the use of moisture dressings. In general, wound dressing provides wound protection, remove excess exudates its may have anti-microbial properties, have high permeability to oxygen, and are easily removed from the wound site (Zeng *et al.* 2018). There have been some experiments with polysaccharide beads, which are highly hydrophilic and rapidly absorb exudate from the necrotic sloughy mass. Hydrogels have proven to be more effective (Eneroth & van Houtum 2008; Edwards & Stapley 2010) and are now recognised as a standard treatment for necrotic wounds (Edwards 2010). Hydrogel compositions vary from sodium carboxymethylcellulose aqueous based-gel (D’Hemecourt 1998) and hydrogel wound dressings (Dumville *et al.* 2013) to immunomodulating hydrogel with bacteriostatic agents (Dumville *et al.* 2017). New materials for wound dressings are continuously being developed and include protein-based dressings (Vasconcelos & Cavaco-Paulo 2011), or the combination of natural origin components with nanostructured materials (Andreu *et al.* 2015). In general, all these are gentle debriders as they keep the affected area moist so that natural enzymatic reactions can take place (Edwards, 2010; Zeng *et al.* 2018) though they are slow, require multiple dressing applications, and may not be effective for patients with compromised immune system (McCallon *et al.* 2014).

### Enzymes in Debridement

It is known that wound debridement requires multiple proteinase specificities to degrade the eschar and that these enzymes are supplied by inflammatory leucocytes infiltrating the eschar from the wound bed (Thomas *et al.* 1999). Reepithelialisation is also dependent on the activity of different proteolytic enzymes. During the initial re-epithelialisation of cutaneous wounds, the migrating epidermis reaches the granulation tissue and then dissects the fibrin clot from viable tissue (Clark 1997; Kubo *et al.* 2001). This process is partially mediated by fibrinolytic activity necessary for the wound epidermis to cleave fibrin from its migratory pathway (Rømer *et al.* 1996) and metalloproteinases that are important for keratinocyte penetration under the fibrin clot (Saarialho-Kere *et al.* 1993: 1994). Trypsin activity is known to favour fibrocyte differentiation, while proteases with other specificities such as pepsin, endoprotease GluC, and chymotrypsin do not promote this process (White *et al.* 2013). Matrix metalloproteinases are also crucial in the synthesis, degradation, cross-linking, and reorientation (i.e. remodeling) of collagen during the last stages of healing (Satish & Kathju 2010; Robichaud *et al.* 2011). Thus, natural debridement and subsequent re-epithelialisation can be referred to as enzymatic processes.

One method of debridement is the use of the insect maggot (*Lucilia sericata*), referred to as larval therapy or biologic debridement. In this process, the maggot breaks down biofilms of various bacteria and their actions seem to be limited to the necrotic wound while sparing the healthy tissue, thus it is a selective method. This method was widely practiced in the past and is now gaining renewed attention (Eneroth & van Houtum 2008; Gottrup & Jørgensen 2011). There is now strong evidence for the biochemical mechanisms underlying the success of this method (Smith *et al.* 2006). Maggot excretions and secretions contain allantoin, sulfhydryl radicals, calcium, cysteine, glutathione, embryonic growth stimulating substance, growth stimulating factors for fibroblasts, carboxypeptidases A and B, leucine aminopeptidase, collagenase, and proteases (trypsin, chymotrypsin, metalloproteinase and aspartyl proteinase) (Gupta 2008). These maggot proteinases hydrolyse fibrin clots, and its chymotrypsin-like fraction degrades extracellular matrix components such as fibronectin, laminin and acid-solubilised collagen types I and III (Chambers *et al.* 2003). Due to the relevance of chymotrypsin activity for maggot debridement, a chymotrypsin I from maggot was manufactured as a recombinant enzyme and proved to digest chronic wound eschar *ex vivo* (Telford *et al.* 2011). Although this chymotrypsin was inhibited by alpha-2-macroglobulin, it was unaffected by main inhibitors in wound eschar (alpha-1-antichymotrypsin and alpha-1-antitrypsin), which is different from the effect of these inhibitors on the mammalian equivalent, alpha-chymotrypsin. Maggot chymotrypsin proved to cleave the major proteins from slough/eschar from venous leg ulcers better than chymotrypsins from human and bovine sources (Telford *et al.* 2010). Other reports support that maggot secretions also stimulate wound healing by promoting different cellular processes such as activation of fibroblast migration, angiogenesis, and production of growth factors within the wound (Nigam & Morgan 2016). The secretions of other insects have also been evaluated as debriders; secretions from the larvae of the blowfly *Calliphora erythrocephala* digested experimental rat skin burn eschar *in vivo* and *in vitro*. Interestingly, there was no evidence of chymotrypsin, elastase, or collagenase in these secretions but enzymes with trypsin, leucine aminopeptidase, and carboxypeptidases A and B activities were found (Vistnes *et al.* 1981).

Some methods use enzymes directly over the wound and are known as enzymatic debridement (Carson *et al.* 2003; Hwang & Ivy 2006; Ramundo & Gray 2009). Although the clinical experience suggests that combined therapy (e.g. initial surgical debridement followed by serial enzymatic debridements) is effective for many patients with chronic, indolent, or nonhealing wounds, enzymes may be used as the primary technique in certain cases when surgical debridement is not a good option due to bleeding disorders (Ramundo & Gray 2008), diabetes mellitus (Edwards & Stapley 2010), or complicated anatomy in the case of deeply burned hands (Krieger *et al.* 2011). Enzymatic debridement such as collagenase ointment and papain-urea-based ointment are more effective for debridement of necrotic tissue from pressure ulcers, leg ulcers, and partial-thickness burn wounds, than standard methods (Marazzi *et al.* 2006, Ramundo & Gray 2009; Shi & Carson 2009; Shi *et al.* 2010; Patry & Blanchette 2017). Collagenase (i.e. *Clostridium* collagenase) exhibits activity in the pH range found in most chronic wounds (Shi *et al.* 2011) and achieves selective debridement by digesting denatured collagen in eschar while sparing non-necrotic tissues (Shi & Carson 2009). Also, a formulation containing streptokinase and streptodornase digests fibrin, collagen and elastin, which are found in the necrotic exudates of wounds. Vibriolysin, a proteolytic enzyme secreted by the marine microorganism *Vibrio proteolyticus* has been evaluated *in vitro* as an enzymatic debridement agent to successfully hydrolyse proteinaceous components of eschar (e.g. fibrin, elastin, collagen) (Durham *et al.* 1993). Also, a bromelain-based preparation decreased the wound area and skin-graft use (Krieger *et al.* 2011) and accelerated re-epithelialisation as well (Singer *et al.* 2011). Bromelain is a mixture of enzymes such as phosphatase, glucosidase, peroxidase, cellulase and escharase, from the fruit or stem of pineapple (Pavan *et al.* 2012). In deep burned hands, the use of the bromelain-based NexoBrid^®^ is promising regarding handling and duration of the treatment, efficiency and selectivity of debridement, healing potential and early rehabilitation (Schulz *et al.* 2017). Other enzyme formulations have also been applied commercially to the debridement of ulcers including bovine trypsin, bovine or human plasmin, and subtilisin (Aehle 2004; McCallon *et al.* 2014). Some of these enzymes have also been tested in combination with antimicrobial dressings (Shi *et al.* 2010). From these methods, collagenase treatment is one of the most commonly used method in clinical practice in conjunction with topical antibiotics. However, available clinical trials present a high risk of bias and more research and adequate reporting of adverse events has been warranted (Patry & Blanchette 2017).

## CRUSTACEAN PROTEASES AND THEIR DEBRIDEMENT POTENTIAL

### Proteases from Cold-adapted Crustacean Species

Marine enzymes evaluated for debridement applications are from cold-adapted species (Fornbacke & Clarsund 2013). Enzyme mixtures from krill (a small crustacean from cold waters) were tested and proved to be more active than other commonly available proteases used for wound debridement (Mekkes *et al.* 1997; Mekkes *et al.* 1998; Hellgren *et al.* 1986; Anheller *et al.* 1989; Campell *et al.* 1987; Westerhof *et al.* 1990; Hellgren *et al.* 1991). The predominant activity in the protease extract from krill is trypsin-like activity associated with three serine proteinases, in addition to two carboxypeptidases, A and B (Anheller *et al.* 1989). There are also some krill enzymes with wide specificity (crustacean serine collagenolytic enzymes or brachyurins Ia). Type Ia brachyurins possess trypsin-, chymotrypsin- and elastase-like activities in addition to their ability to hydrolyse collagen (Barret *et al.* 1998). These cold-adapted enzymes have low activation energy and high catalytic efficiency (kcat/Km). The structural features of trypsins and other enzymes from cold-adapted organisms, including non-crustacean species, determining their high catalytic efficiency have been discussed extensively (Adekoya *et al.* 2006; Papaleo *et al.* 2008). An increased structural flexibility is thought to reduce the activation energy necessary for generating reaction intermediates, resulting in a more efficient substrate turnover, although differences in catalytic efficiency between bovine and cold-adapted salmon trypsins are thought to result from differences in Km (Sekizaki *et al.* 2000), probably due to variation in the electrostatic potential of the S1-binding pocket (Gorfe *et al.* 2000). One of the krill enzymes, referred to as euphauserase, has been produced recombinantly in *P. pastoris* for use in debridement (Kristjansdottir & Gudmundsdottir 2000). Similar efforts were made with cold-adapted cod trypsin. A clinical study using an hydrogel formulation containing cod trypsin on pressure wounds revealed that it was of superior efficacy for wound healing compared to conventional treatments (Mangioli 2004). In addition to its role in debridement, cod trypsin exhibits anti-pathogenic effects (Gudmundsdóttir *et al.* 2013).

However, one limitation for the use of cold-adapted enzymes is their poor thermal stability. The activity of cod trypsin is maintained for longer periods at 15°C than at higher temperatures (Stefansson *et al.* 2010). Another feature of this enzyme (and that from krill) is its susceptibility to autolysis, with several autolytic cleavage sites (Stefansson *et al.* 2010). It has been proposed that the thermal inactivation of cod trypsin, involving unfolding and autolysis, limits the lifespan of the enzyme in the wound bed, and may decrease the risk of harm the viable tissue (Gudmundsdóttir *et al.* 2013). However, efforts have been made to increase thermal stability of both cod trypsin (Gudmundsdóttir & Pálsdóttir 2005) and krill euphauserase (Benjamin *et al.* 2001; Gudmundsdóttir & Pálsdóttir 2005) by site directed mutagenesis, and by covalent chemical modification with oxidised sucrose polymer in the case of the cod enzyme (Venkatesh *et al.* 2005). However, thermal stability for the krill enzyme only increases from 35°C to 40°C after site directed mutagenesis (Benjamin *et al.* 2001). In spite of this, it was considered that the best resulting mutant was the one that combines a long lasting high proteolytic activity (one single mutation provided around 25 times more proteolytic stability) with thermal instability, because the latter allows a tighter control of the enzymatic activity during biomedical application (Olivera-Nappa *et al.* 2013**)**. However, others have proposed that the thermal instability of cold-adapted trypsins represents a drawback for their practical use, taking also into consideration the prospects for its production (de la Cruz *et al.* 2018). In the case of the krill enzyme, an analysis of a structural model suggested that two residues of loop D (analogous to the autolysis loop of trypsins) might be targets for autolysis and thus, changes were incorporated at these positions; the mutant was more stable against autolysis than the wild-type form of the enzyme during the production of the recombinant (Gudmundsdóttir & Pálsdóttir 2005).

Wide specificity proteases from other crustaceans have been also evaluated as debrider agents. It was reported that the red king crab (*Paralithodes camtschaticus*) collagenase has higher proteolytic activity toward fibrin clot and necrotic eschar *in vitro* than four enzyme preparations (trypsin/chymotrypsin, and proteases isolated from *Aspergillus terricola*, *Carica papaya* and pseudomonodaceae), and it was tested successfully in a small clinical trial with patients with leg ulcers (Glyantsev *et al.* 1997). This enzyme is also cold-adapted, thus its stability is affected by high temperatures. In addition, many cold-adapted proteolytic enzymes are negatively affected in acid media (Gudmundsdóttir & Pálsdóttir 2005).

### Proteases from Tropical *versus* Temperate Crustacean Species

Enzymes of organisms from warm environments are often more thermally resistant than cold-adapted enzymes (Table 1). This is thought to results from stronger hydrophobic interactions at the inner protein structure and, in some cases, because of differences in the number of disulfide bridges. Cold-zone fish trypsins have lower thermal stabilities, in general less than 30°C–40°C, than temperate and tropical fish trypsins (Kishimura *et al.* 2010). Clear examples for this trend also arise when comparing enzymes from similar crustaceans, even from the same genus, but adapted to different environmental temperature. We have studied trypsins isoenzymes from the tropical lobster *Panulirus argus*. The molecular masses of lobster trypsins range from 35 to 36 kDa. All enzymes have restricted trypsin activity and show the distinctive trypsin preference for Arg over Lys in the P1 position. Optimal pH for most isoforms ranges from 7 to 8, although one trypsin exhibits maximum activity at pH 6–6.5, and this same isoenzyme has double the activity of the other isoforms at pH 5 (Perera *et al.* 2012). While all *P. argus* trypsins are less efficient than those present in some fish (e.g., *Engraulis japonicus*, Ahsan & Watabe 2001; *Oncorhynchus keta*, Toyota *et al.* 2007) and cold-adapted crustaceans (e.g., *Euphausia pacifica*, Wu *et al.* 2008), they are more efficient than bovine trypsin (Rascón *et al.* 2011). The tropical lobster trypsins were stable at 55°C for at least 60 min and the same was observed for chymotrypsin (Perera *et al.* 2012). Conversely, a chymotrypsin from the gastric juice of a temperate lobster from the same genus, *Panulirus interrutpus*, is inactivated after 20 min at the same temperature (Bibo-Verdugo *et al.* 2015). The chymotrypsin from *P. interruptus* showed collagenase activity because it presents the same residues in the S1 binding pocket, as does the brachyurin from *Uca pugilator* (Bibo-Verdugo *et al.* 2015). The same is likely to be true for chymotrypsin from the tropical lobster *P. argus* although functional data are not available.

A trypsin-like enzyme from the blue swimmer crab (*Portunus pelagicus*), adapted to high temperatures, shows only 50% inactivation at 68°C (Dionysius *et al.* 1993). Conversely, in the temperate crab species *Cancer pagurus*, trypsin activity decreased to 30% at 50°C, losing the activity after 10 min at 60°C (Saborowski *et al.* 2004). Although chymotrypsin in this species was more heat resistant than trypsin, the activity decreased towards 60% of the initial value at 50°C (Saborowski *et al.* 2004). Trypsins from two hermit crabs, the temperate *Pagurus bernhardus* and the tropical *Clibanarius striolatus*, also differed in their thermal stability (Dittrich 1992). At 50°C, the protease from the tropical crab does not lose any activity after 120 min whereas after this period, only 9% residual activity was found for the enzyme from the temperate species (Dittrich 1992). At the same temperature, proteases from the gastric juice of another temperate crab, *Cancer pagurus*, show reduced activity by about 60% (trypsin) and 40% (chymotrypsin) in 60 min (Saborowski *et al.* 2004). Another study revealed that digestive alkaline proteases of two cold-water species, the Chilean rock crab (*Cancer edwardsii*) and the southern king crab (*Lithodes santolla*), completely lost their activity after 20 min at 60°C (Bañuelos-Vargas *et al.* 2018). Conversely, recent studies have shown that trypsin and chymotrypsin activities from a related species, the tropical king crab *Maguimithrax spinosissimus*, remain above 80% after 60 min at 40°C (Rodríguez-Viera *et al.* unpublished).

Studies have been also conducted on Penaeids shrimps because of their importance in aquaculture production worldwide. Each shrimp species has its own optimum temperature range in terms of maximum yield during culture. Trypsin from the tropical penaeid shrimp *Litopenaeus vannamei* is stable up to 60°C, with a residual activity of 95%–99% after 15 min (Senphan *et al.* 2015). Chymotrypsin from *L. vannamei* is also thermostable, retaining more than 80% of activity after 60 min at 50°C (Hernandez-Cortes *et al.* 1997). Conversely, the pink shrimp *Farfantepenaeus paulensis* is considered to be suited for culture in sub-tropical and temperate areas, and its trypsin activity is drastically reduced after 15 min at 45°C (Buarque *et al.* 2009). Chymotrypsin activity in this shrimp is also affected at 55°C (Buarque *et al.* 2009). A few other examples are shown in Table 1 with focus on trypsin enzymes. The high thermal stability of digestive proteases from tropical crustaceans would represent an advantage over their cold-adapted or temperate counterparts, as debridement and wound healing proceed better at body temperature in general. It is known that when the temperature of a wound falls below body temperature, healing can be significantly delayed. In fact, some authors have found that wound bed temperatures immediately drop below 33°C after dressing removal, which is the minimal temperature threshold for normal wound healing (McGuiness *et al.* 2004).

On the other hand, tropical lobster (*P. argus*) trypsins are fairly resistant to auto-proteolysis (Perera *et al.* 2008). Auto-proteolytic stability is a feature shared by different crustaceans from both warm and cold environments (Sainz & Córdova-Murueta 2009; Hehemann *et al.* 2008). In shrimps, active trypsin can be recovered from feces (Córdova-Murueta *et al.* 2003) and this is likely to be true in other crustaceans. The use of proteases that are both thermostable and resistant to proteolysis in debridement and related treatments is advisable in terms of prolonged therapeutic effects and the possibility of decreasing the frequency of dressing changes due to wound temperature stability considerations (McGuiness *et al.* 2004).

Another issue related with the use of proteases in debridement and similar applications is the occurrence of protease inhibitors in the intended place of action. In the cold-adapted hermit crab *P. bernhardus,* soybean trypsin inhibitor (SBTI) suppresses the activity to about 5%, whereas in the tropical hermit crabs *C. striolatus* only to 30% (Dittrich 1992). However, differences in the susceptibility to inhibitors between tropical *versus* temperate crustacean proteases are not clear (Perera *et al.* 2015). Inhibitor affinity is highly dependent on the structural feature of enzymes. Three-dimensional homology models developed for trypsins from the lobster *P. argus* anticipated structural differences among the iso-enzymes (Perera *et al.* 2010; 2015) and with trypsin from crayfish (Fodor *et al.* 2005) in important regions for inhibitor binding (Fodor *et al.* 2005; Molnár *et al.* 2013; Díaz-Mendoza *et al.* 2005). Indeed, the trypsins from *P. argus* differ in their susceptibility to SBTI, with inhibition ranging from 92% to 100% when the inhibitor was tested at a concentration similar to that of Human alpha-1 antitrypsin in human plasma (Perera *et al.* 2012).

It is known that alpha-1-antitrypsin is degraded and non-functional in chronic wounds but intact and functional in acute wounds, and that the inhibitor protects fibronectin (key component of the provisional matrix in skin wounds) from degradation by wound fluid enzymes (Rao *et al.* 1995) and improves healing (Rao *et al.* 1995; Cathomas *et al.* 2015). In fact, a treatment has been developed that uses alpha-1-antitrypsin for the preparation of a wound dressing composition for the treatment of chronic wounds (Grady *et al.* 2003). Also, it is known that non-healing leg ulcers have persistently elevated levels of proteases that prevent healing, which degrade growth factors and disrupt the balance between tissue breakdown and repair (McCarty & Percival 2013). For example, matrix metalloproteinase (MMP) activity was observed to be 30-fold higher in chronic wounds than in acute wounds (Trengove *et al.* 1999). Indeed, high levels of MMP activity and low levels of MMP inhibitor impair wound healing in chronic pressure ulcers (Ladwig *et al.* 2002). Similar results were reported for elastase activity (Yager *et al.* 1997). Accordingly, a protease-modulating matrix treatment, which removes proteases from the wound fluid, has been evaluated on venous leg ulcers, although its value is still unclear (Westby *et al.* 2016). Altogether, these results suggest that the effects of crustacean proteases should be different in chronic and acute wounds, or at different stages of healing, and this merits further examination. The ability of digestive proteases from tropical crustaceans to digest human fibronectin and growth factors also deserve investigation as part of the evaluation of their biomedical potential in wound healing.

To the best of our knowledge, no digestive protease from tropical crustaceans has been evaluated for debridement and wound healing despite the fact that chymotrypsin from the tropical shrimp *L. vannamei* is highly homologous (77%) to crab collagenase (Sellos & Van Wormhoudt 1992). Type Ib brachyurins (crustacean chymotrypsins) share the specificity of type Ia brachyurins, but with a drastic reduction of trypsin activity. Also, collagenolytic serine proteases from *L. vannamei* are able to digest native porcine type I collagen (Burgos-Hernández *et al.* 2005) as reported for chymotrypsin from another shrimp species, *Penaeus californiensis* (Navarrete-del-Toro *et al.* 2015). Trypsins from the warm water shrimp *Penaeus monodon* also have collagenolytic activity in addition to the typical specificity of trypsin (Lu *et al.* 1990), although a further study suggested that this may occur by activation of procollagenase in the native collagen (Chen *et al.* 1991). Also, one trypsin-like protease from *P. argus* has amino acid substitutions in the vicinity of the active site that may impair the access of bulky residues to the S1 site and make the pocket more hydrophobic, which probably confers elastase-like activity to this enzyme (Perera *et al.* 2010). In addition, as prolonged inflammation characterises chronic wounds, and trypsin from fish are able to degrade inflammatory cytokines (Gudmundsdóttir *et al.* 2013), it is plausible that trypsins from tropical crustaceans may have a similar anti-inflammatory effects.

Altogether, these results indicate that we are far from understanding the true potential for biomedical applications of proteases from tropical crustaceans. In this regard, the identification of potentially useful enzymes according to the thermal habitat and feeding behaviour of the source may direct the discovery of enzymes with desired specificities and physical and chemical properties. For instance, digestive enzymes of tropical crustaceans cleaner organisms (and fish) that feed on dead skin of other fish in cleaning stations (Floeter *et al.* 2007) would be worthy to examine, given their preference for skin proteins as substrates. Examples of these organisms include cleaner shrimps (e.g. *Lysmata* spp.*, Periclimenes* spp*., Stenopus* spp*.*) and fishes such as wrasses and gobies, but global cleaner diversity was recently estimated to be 208 fish species from 36 families and 106 genera, and 51 shrimp species from six families and 11 genera (Vaughan *et al.* 2017). Although not naturally feeding on skin, the use in spa skin treatments of fish such as the toothless *Garra rufa*, which feed on dead skin tissue under conditions of feed scarcity, is interesting (Schets *et al.* 2015). These organisms would be sources of enzymes with different biomedical applications and in particular with debridement and/or wound healing properties.

## CONCLUSION AND OPEN ISSUES

Marine organisms contain a great variety of digestive enzymes that have evolved to fulfill their wide variation in feeding habitats, natural substrates in feed, and environmental conditions; thus, they have accumulated millions of years of sequence evolution and structural refinements to achieve this functionality. Unfortunately, few genomic resources are available for marine crustaceans, hindering the screening of enzymes for biomedical application. However, the examination of potentially useful enzymes according to feeding behaviour and thermal habitat of the source may direct the discovery of enzymes with desired specificities and physical and chemical properties. This pathway is far from being fully exploited.

The thermal instability of cold-adapted enzymes provides benefits for the food industry because they can be selectively inactivated by mild heat input, which results in the protection of the food being processed (Klomklao 2008; Kuddus 2018). However, this feature remains a drawback for their use in debridement and similar applications. Trypsin and other proteases from tropical crustaceans may have similar specificities to those exhibited by proteases from cold-adapted fish and crustaceans for which wound healing properties have been demonstrated. Moreover, tropical crustaceans have more thermally resistant proteases, and also exhibit autoproteolytic stability; thus, they may have advantages for applications such as debridement and other topical treatments. Also, there are possibilities to extract these enzymes in an environmentally friendly way from the many tons of fishery and aquaculture wastes from both fish and crustaceans. However, in the case of crustaceans, the extraction and applications of chitin, chitosan, and their derivatives in pharmaceuticals and biomedicines have received most of the attention (Nguyen *et al.* 2017) up to now. There is a potential for the biomedical use of proteases and other enzymes from tropical crustaceans, but most studies have been focused in cold-adapted and temperate species.

## Figures and Tables

**Table 1:** Thermal properties of crustacean trypsins.

Species	Climate zones	Optimum temperature (°C)	Thermal stability (°C)	References
*Panulirus argus* (Lobster)	Tropical	60	55	Perera *et al*. (2012)
*Thenus orientalis* (Lobster)		–	–	Johnston *et al*. (1995)
*Fenneropenaeus indicus* (Shrimp)		45	35–40	Honjo *et al*. (1990)
*Litopenaeus vannamei* (Shrimp)		60^a^	60^b^	^a^ Sainz *et al*. (2004) ^b^ Senphan *et al*. (2015)
*Penaeus monodon* (Shrimp)		55–65	50	Jiang *et al*. (1991)
*Callinectes bellicosus* (Crab)		50	40–50	Díaz-Tenorio *et al*. (2006)
*Callinectes arcuatus* (Crab)		50	40–50	Díaz-Tenorio *et al*. (2006)
*Callinectes sapidus* (Crab)		70	50	Dendinger & O’Connor (1990)
*Portunus pelagicus* (Crab)		60	65	Dionysius *et al*. (1993)
*Scylla serrata* (Crab)		40	60	Serrano (2015)
*Maguimithrax spinosissimus* (Crab)		60	40	Rodríguez-Viera *et al*. (unpublished)
*Clibanarius striolatus* (Hermit crab)		50	50	Dittrich (1992)
*Macrobrachium rosenbergii* (Crayfish)		55	40	Sriket *et al*. (2012)
*Macrobrachium amazonicum*		65	55	da Silva Santos *et al*. (2014)
*Tisbe biminiensis* (Copepod)		55	50	França *et al*. (2010)
*Farfantepenaus paulensis* (Shrimp)	Subtropical	45	40	Buarque *et al*. (2009)
*Procambarus clarkia* (Crayfish)		55^a^ 45^b^	45^a^ 45^b^	^a^ Kim *et al*. (1992) ^b^ Guizani *et al.* (1992)
*Panulirus interruptus* (total alkaline protease) (Lobster)	Subtropical/Temperate	50	40	Celis-Guerrero *et al*. (2004)
*Pagurus bernhardus* (Hermit crab)		45	40	Dittrich (1992)
*Lithodes santolla* (total alkaline protease) (Crab)		60	15	Bañuelos-Vargas *et al*. (2018)
*Cancer edwardsii* (Crab)		60	45	Bañuelos-Vargas *et al*. (2018)
*Cyrtograpsus angulatus* (Crab)	Temperate	45	–	Michiels *et al*. (2017)
*Cancer pagurus* (Crab)		–	30	Saborowski *et al*. (2004)
*Paralithodes camtschaticus* (Crab)	Temperate/Polar	55	–	Rudenskaya *et al.* (1998)
*Euphausia pacifica* (Krill) (Euphausid)	Polar	40–50	20–35	Wu *et al*. (2008)
